# ENCAPP: elastic-net-based prognosis prediction and biomarker discovery for human cancers

**DOI:** 10.1186/s12864-015-1465-9

**Published:** 2015-04-03

**Authors:** Jishnu Das, Kaitlyn M Gayvert, Florentina Bunea, Marten H Wegkamp, Haiyuan Yu

**Affiliations:** Department of Biological Statistics and Computational Biology, Cornell University, 335 Weill Hall, Ithaca, NY 14853 USA; Weill Institute for Cell and Molecular Biology, Cornell University, Ithaca, NY 14853 USA; Tri-Institutional Training Program in Computational Biology and Medicine, New York, NY 10065 USA; Department of Statistical Science, Cornell University, Ithaca, NY 14853 USA

**Keywords:** Cancer genomics, Gene expression, Protein interaction network, Prognosis prediction, Elastic net

## Abstract

**Background:**

With the explosion of genomic data over the last decade, there has been a tremendous amount of effort to understand the molecular basis of cancer using informatics approaches. However, this has proven to be extremely difficult primarily because of the varied etiology and vast genetic heterogeneity of different cancers and even within the same cancer. One particularly challenging problem is to predict prognostic outcome of the disease for different patients.

**Results:**

Here, we present ENCAPP, an elastic-net-based approach that combines the reference human protein interactome network with gene expression data to accurately predict prognosis for different human cancers. Our method identifies functional modules that are differentially expressed between patients with good and bad prognosis and uses these to fit a regression model that can be used to predict prognosis for breast, colon, rectal, and ovarian cancers. Using this model, ENCAPP can also identify prognostic biomarkers with a high degree of confidence, which can be used to generate downstream mechanistic and therapeutic insights.

**Conclusion:**

ENCAPP is a robust method that can accurately predict prognostic outcome and identify biomarkers for different human cancers.

**Electronic supplementary material:**

The online version of this article (doi:10.1186/s12864-015-1465-9) contains supplementary material, which is available to authorized users.

## Background

The genetic complexity of cancer and its widely varying etiology and outcome make it extremely difficult to treat. It has been realized that rather than being a single disease, different cancers have widely diverse molecular bases [1,2]. There has been a tremendous amount of effort in the literature to understand molecular signatures underlying cancer [1]. A significant number of these efforts have been informatics-based approaches that try to leverage genomic information such as expression alterations, mutations in genomes, copy number changes and epigenetic modifications to elucidate the mechanistic basis of cancer [3]. Global collaborative research endeavors such as The Cancer Genome Atlas (TCGA) [4] and the International Cancer Genome Consortium (ICGC) [5] are trying to assimilate these genome-scale datasets for different kinds of cancers across many countries.

One of the key challenges has been to use genomic information to understand the basis for different outcomes for the same cancer. However, this has been difficult because it is unclear as to which parameters contain the most information regarding disease outcome. One of the first attempts at predicting cancer prognosis using genome-scale transcriptomic datasets was undertaken by van de Vijver et al. [6]. Using microarrays, they obtained tissue-specific gene-expression profiles for breast cancer patients. They then clustered these expression profiles and correlated them with prognostic outcome to identify a 70-gene ‘prognosis profile’ for breast cancer. One of the key limitations in using only expression datasets to predict cancer prognosis is the assumption of independence between genes in hypothesis testing. The protein products encoded by these genes are not independent but part of a complex interactome network. The dependencies of this network have been shown to be of great importance in understanding the genetic and molecular bases of disease [7-11]. Chuang et al. [12], Taylor et al. [13] and Wu and Stein [14] used a functional interactome network to predict breast cancer prognosis. Recently, Hofree et al. reported a network-based stratification approach that can use somatic mutations to predict cancer subtypes [15]. However, their method is primarily designed to work with mutation data and is less accurate for expression data [15]. Given the much wider availability of expression datasets as compared to whole genome or exome sequences, it is of paramount importance to have a robust method that can use gene expression to accurately predict prognosis across different types of cancer. To this end, in this manuscript, we report ENCAPP, an elastic-net-based cancer prognosis prediction method. We use tissue-specific gene expression data from patients along with the reference human protein interaction network to construct a regression model that can predict disease outcome for breast, colon, rectal, and ovarian cancers. Our approach outperforms previous methods in terms of accuracy of prognosis prediction. Moreover, ENCAPP can also accurately identify genes that can serve as prognostic biomarkers for different cancers.

## Results and discussion

### ENCAPP – a schematic

**Figure 1:**
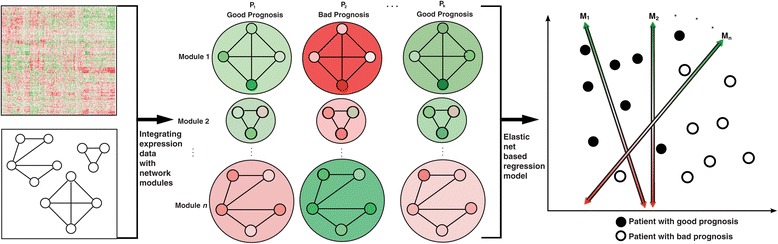
**Schematic of ENCAPP.** ENCAPP begins by overlaying tissue-specific gene expression data with the reference interactome network. Modules that have significant differential co-expression between patients with good and bad prognosis are used to build a regression model that can predict prognostic outcome.

A reference high-quality human protein interactome was constructed as described earlier [16]. Our interactome comprises a total of 42,604 binary and co-complex interactions among 9,985 proteins. We include both kinds of interactions as they capture orthogonal layers of information – binary interactions represent direct contacts between two proteins, while co-complex associations capture co-membership of a protein complex. This network is clustered into different functional modules. We overlay tissue-specific gene expression data from cancer patients onto these functional modules to generate ‘expression modules’. We then identify ones that are differentially expressed between patients with good and bad prognosis (Figure 1). We use the expression modules that show the maximum difference between the prognostic outcome classes as decision boundaries to build a regression model that can predict disease prognosis (Figure 1b). Our regression approach attempts to estimate the conditional probability of having good or bad prognosis given the patient’s expression modules.

Since the data is inherently high dimensional (i.e., the number of expression modules is greater than the number of patients), ordinary least squares regression cannot be used and a regularization term is essential (see Methods). While ridge regression (L2 regularization term) [17] uses all input variables to fit the model, the least absolute shrinkage and selection operator (LASSO, L1 regularization term) [18] attempts to find the most optimal sparse fit. Ridge regression can lead to inflated variance but has low bias, while LASSO can have high bias but ensures low variance. To optimize the bias-variance tradeoff, the elastic net [19,20] has been proposed and is our choice of regression model (see Methods).

### Prognosis prediction using differentially expressed functional modules

**Figure 2:**
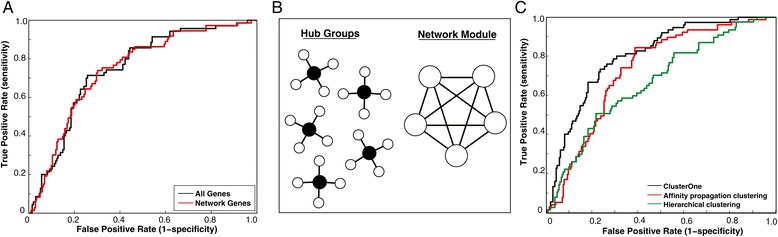
**Integrating gene-expression data with protein interactome networks. (A)** Receiver operating characteristic (ROC) curves for prognosis prediction using expression data alone. **(B)** Illustration of hub groups and networks modules. **(C)** ROC curves comparing the performance of three module-detection algorithms – hierarchical clustering, affinity propagation clustering and ClusterOne.

We first examined expression data from a cohort of breast cancer patients [6]. Here, prognosis was defined as five-year disease-free survival. Using five-fold cross validation, we first measured prognosis prediction accuracy using only expression values from all genes and found it to be a suboptimal predictor (median AUC = 0.747, 95% CI for AUC = 0.743-0.751 Figure 2A, see Methods). Since proteins carry out their function by interacting with other proteins, we then used only expression values from genes whose corresponding proteins have at least one known interaction to predict prognosis. This did not significantly alter performance (median AUC = 0.745, Figure 2A). Taylor et al. used hub groups as a measure of network topology, however we choose modules for two reasons (Figure 2B). First, hub groups only include interactions between the hub protein and its interactors, not those between the interactors themselves. Thus, modules contain more information. Second, in Taylor et al’s model, each protein is assigned to one and only one hub group. However, since network modules can be overlapping [21,22], the same protein may be assigned to multiple modules if it has multiple functions. Since numerous proteins carry out biological functions in a pleiotropic fashion, our approach captures such relationships while hub groups do not.

To identify functional modules, we tried three separate algorithms – hierarchical clustering [23], affinity propagation clustering [24] and ClusterOne [25]. We constructed modules from all three algorithms using default parameters (the module creation is independent of any expression data). Using expression values from the van de Vijver dataset, we used modules generated by all three algorithms to construct expression modules and used them to predict prognosis. We find that ClusterOne has the best performance (Figure 2C; see Methods). One possible reason for this is that the protein interactome network is binary (1 corresponding to an interaction between two proteins, while 0 corresponds to no interaction between the two proteins) and sparse. Thus, the number of discrete values (equal to 1 + the graph diameter) the graph distance used for hierarchical clustering can take is limited. Affinity propagation clustering is more suited to identifying hub-group-like topological structures as hubs fit the definition of exemplars. On the other hand, ClusterOne was designed to identify functional modules that capture pleiotropic relationships. Thus, ClusterOne was used for all further analyses.

**Figure 3:**
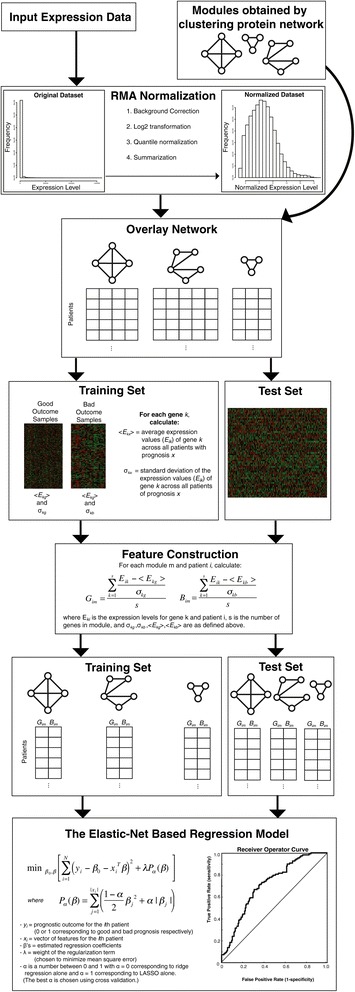
**Flowchart illustrating the different steps in ENCAPP.** The inputs to ENCAPP are RMA-normalized expression data and modules from a reference human protein interactome network. These are then combined into features that are input to an elastic-net based regression model. The performance of the model is evaluated using cross-validation.

**Figure 4:**
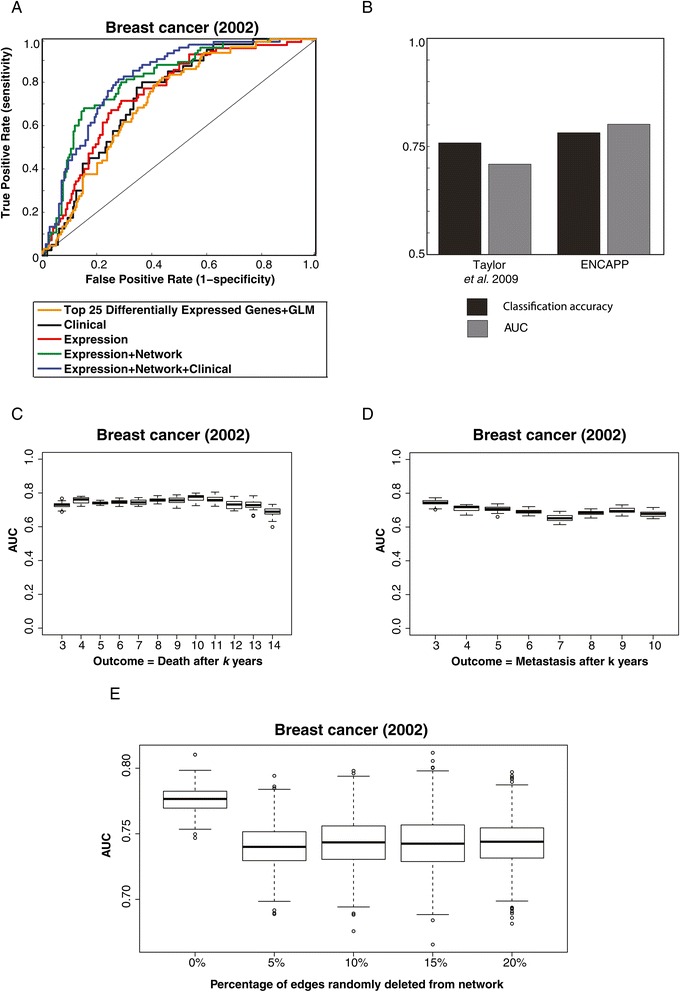
**Predicting breast cancer prognosis using differentially expressed functional modules. (A)** ROC curves for prognosis prediction of patients in the breast cancer (2002) dataset using clinical data alone, expression data alone, expression data with the protein network and all 3 datasets together. **(B)** Comparison of the performance of ENCAPP with Taylor et (values shown are those obtained in the absence of clinical information). **(C)** Boxplots showing performance of ENCAPP at different right censoring cutoffs *k* used for determining prognostic outcome: for each boxplot, good prognosis is defined as survival for > =*k* years and bad as death within *k* years. **(D)** Boxplots showing performance of ENCAPP at different right censoring cutoffs *k* used for determining prognostic outcome; here a different outcome definition is used: for each boxplot, good prognosis is defined as no metastasis for > =*k* years and bad as metastasis within *k* years. **(E)** Boxplots showing performance of ENCAPP using random networks that have 5%, 10%, 15% and 20% of the total edges in the original network randomly removed.

We then explored the contribution of the three different datasets – clinical covariates, gene expression and the protein network to predicting prognosis. Figure 3 presents a flowchart of how these datasets are combined in our ENCAPP algorithm. We find that expression and network in combination are the most informative (Figure 4A, median AUC = 0.777; 95% CI for AUC = 0.773-0.780; *P* < 10^−3^; Additional file 1: Table S1) and the addition of clinical data only marginally improves the performance (Figure 4a, AUC = 0.786; 95% CI for AUC = 0.783-0.789; *P* < 10^−3^; Additional file 1: Table S1). ENCAPP also performs much better than an approach that just uses differential expression; we trained a generalized linear model with differentially expressed genes selected using the LIMMA package [26] and found that the median AUC is 0.685, significantly lower than ENCAPP (*P* < 10^−3^). These results confirm that using interaction dynamics, a combination of gene expression data with the topological structure of the network, is a key predictor of prognosis. Our results also confirm that ENCAPP will work efficiently even in the absence of clinical information, which can be hard to collect and thus is often unavailable. Furthermore, while we used ‘death’ as the outcome variable for the prognosis prediction described above, we find that it is robust to using other variables as outcome labels (Additional file 2: Supplementary Notes).

To compare the performance of our method to previous attempts, we first compared our classification accuracy (i.e., the fraction of patients for which we were able to accurately predict prognosis, see Methods) and AUC to Taylor et al. (Figure 4B). Using expression data in conjunction with the protein network, ENCAPP achieves a median AUC of 0.777, significantly higher than the value of 0.71 reported by Taylor et al (*P* < 10^−3^). We then compared the performance of ENCAPP to that of Chuang et al. At a fixed sensitivity of 90%, ENCAPP has a significantly higher accuracy (75.1% vs 70.1%, *P* = 0.025). Finally, we compared ENCAPP to the results reported by Wu and Stein. Since they do not directly report ROC curves, we adopted a slightly different approach for this comparison. We trained a generalized linear model (GLM) using expression values from van de Vijver for the significant modules identified by them and attempted to predict prognosis for the Wang dataset. We found that the median AUC is 0.510. We then used the same modules and constructed the features that ENCAPP uses to train a GLM. The median AUC goes up to 0.561, significantly higher (*P* < 10^−3^) than the earlier median AUC.

We then sought to assess the changes that cause the performance boost over previous methods. We used ENCAPP on an experimentally verified subset of the Ophid interactome used in the Taylor et al. study. We obtained a median AUC of 0.750, which is significantly higher (*P* = 0.040) than the AUC of 0.71 obtained by them. This confirms that a large portion of the increase in performance is solely due to the core methodology underlying ENCAPP – our approach captures more information regarding the topology of the protein interactome than Taylor et al because of the differences in hub groups and modules outlined earlier. The rest of the increase is due to a higher quality protein network used in our study. The improvement in the protein network can be attributed to two factors – a methodological enhancement: we employ a series of stringent filtering steps [16] to identify a set of high-quality interactions and an increase in the available data. Thus, ENCAPP is a robust and reliable method that combines expression data with protein network modules to accurately predict cancer prognosis; it works efficiently even in the absence of clinical data.

### Robustness of ENCAPP

Is ENCAPP robust to changes of the response variable or the incompleteness of the reference protein network? To systematically test this, we first focused on how the performance of ENCAPP changes when the response variable is altered. For the van de Vijver dataset, he outcome variable (survival) is right censored, i.e., if a patient survives for > =5 years, she is considered to have good prognosis, else bad prognosis. To test the robustness of ENCAPP to the right censoring cutoff, we varied it from 3–14 years i.e, a patient is defined to have good prognosis if she survived for > =*k* years, where *k* varies from 3 to 14. We find that ENCAPP performs consistently well for all values of *k* (Figure 4C), with the highest median AUC being 0.778 and the lowest median AUC being 0.730. This confirms that ENCAPP is robust across a wide range of cutoff values for right censoring.

To further validate the robustness of ENCAPP to alternate definitions of prognosis, we modified the outcome definition. We defined a patient to have a good prognosis, if she does not have metastases for > = *k* years, where *k* varies from 3 to 10. Here too, ENCAPP performs consistently well (Figure 4D), with the highest median AUC being 0.744 and the lowest median AUC being 0.652, confirming that it is also robust across prognosis definitions.

To address the robustness of ENCAPP to incompleteness of the protein network, we generated sets of 50 random networks for each of the following scenarios: 5%, 10%, 15% and 20% of the total edges randomly removed. We then generated modules for all these random networks using the same ClusterOne parameters as the original network. We then re-calculated the performance of ENCAPP on the van de Vijver dataset for each of these networks with a certain fraction of the edges removed. We find that ENCAPP still performs well, with median AUCs of 0.744, 0.740, 0.743 and 0.742 at 5%, 10%, 15% and 20% edge deletions respectively (Figure 4E), confirming that it highly robust to network incompleteness.

### Pan-cancer prognosis prediction

**Figure 5:**
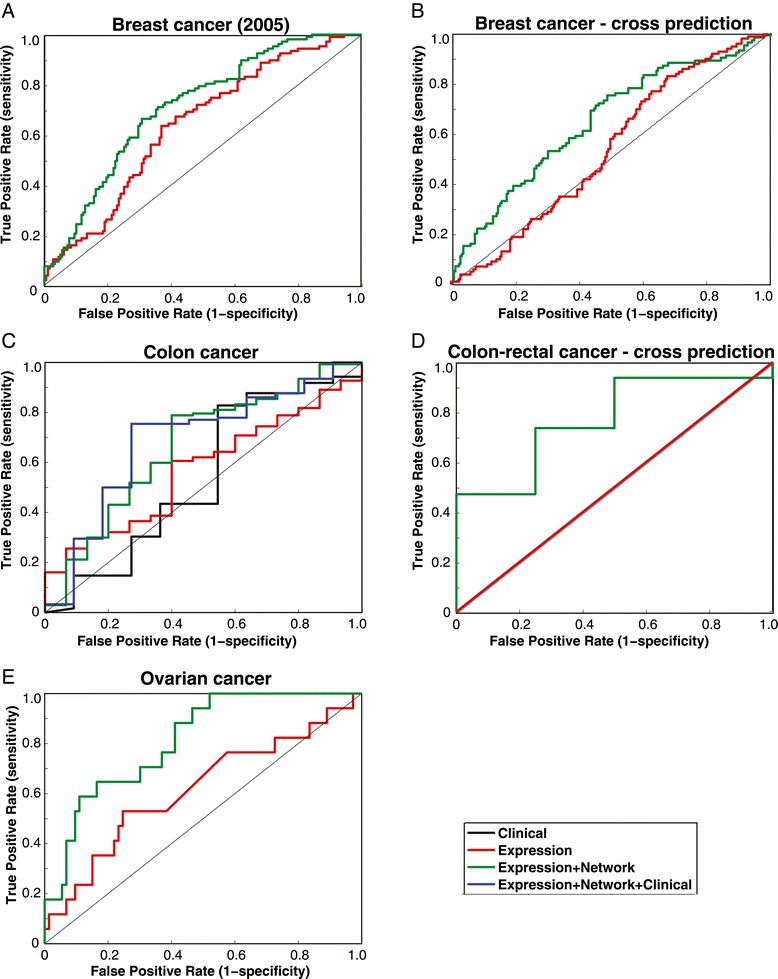
**Prognosis prediction for different cancer types and subtypes. (A)** ROC curves for prognosis prediction of patients in the breast cancer (2005) dataset using expression data alone and expression data with the protein network. **(B)** ROC curves for prognosis prediction of patients in the breast cancer (2002) dataset using data from the breast cancer (2005) dataset. **(C)** ROC curves for prognosis prediction of patients in the colon cancer dataset using clinical data alone, expression data alone, expression data with the protein network and all 3 datasets together. **(D)** ROC curves for prognosis prediction of patients in the rectal cancer dataset using data from the colon cancer dataset. **(E)** ROC curves for prognosis prediction of patients in the ovarian cancer dataset using expression data alone and expression data with the protein network.

A major challenge of prognosis prediction algorithms is to make them generically applicable to different human cancers. To examine the applicability of ENCAPP for other cancer types and sub-types, we first used it on a dataset of lymph-node negative breast cancer patients [27]. Although, van de Vijver et al. also examined breast cancer patients, the consensus gene signature identified was very different. Wang et al. stated that the results vary so much “because of differences in patients, techniques, and materials used” [27]. The van de Vijver dataset included node-negative and node-positive patients and women less than 53 years old. Moreover, prognosis for the Wang dataset is defined as metastasis-free survival. However, ENCAPP is still able to accurately predict (median AUC = 0.690; 95% CI for AUC = 0.684-0.695; *P* < 10^−3^; Additional file 1: Table S1) cancer prognosis for these patients (Figure 5A), confirming that its robustness across cancer sub-types.

Another key goal of prognosis prediction algorithms is to be applicable across data collected from different cohorts of patients. To test whether ENCAPP can be trained on a certain dataset and then used to predict outcome for a completely different set of patients, we used the Wang et al. dataset to train the model and then predicted outcomes for the van de Vijver dataset using it. While we originally analyzed the van de Vijver dataset in terms of overall survival, clinical information on metastasis was available. Since, the Wang et al. dataset uses metastasis-free survival as the prognostic outcome, we used this as the outcome for the cross-dataset prediction. ENCAPP was accurate in predicting outcomes (median AUC = 0.649; 95% CI for AUC = 0.649-0.650; *P* = 0.019; Additional file 1: Table S1), showing that our approach is highly robust and successful in incorporating major differences in clinical parameters (Figure 5B). Here too, we perform better than Chuang et al. who report a classification accuracy of 55.8% at 90% sensitivity (for predictions on the Wang dataset using the van de Vijver sub-network markers). ENCAPP achieves a significantly higher classification accuracy of 62.6% at 90% sensitivity (*P* = 0.009).

We then used ENCAPP to analyze other kinds of cancer – a colon cancer [28] and an ovarian cancer [29] expression dataset published by the TCGA. The ovarian cancer dataset that we analyzed consisted of platinum-resistant cancer patients, which occurs in approximately 25% of patients within 6 months of therapy. For each dataset, we looked to see how well our method could predict overall survival. ENCAPP was able to predict prognostic outcome successfully for both colon and ovarian cancer (median AUCs = 0.666 and 0.766 respectively; 95% CIs for AUC = 0.658-0.674 and 0.760-0.771 respectively; *P* = 0.001 and 0.097 respectively; Additional file 1: Table S1) confirming that it works robustly across different cancers (Figures 5C, 5E).

Finally, we tried using ENCAPP to predict prognosis across cancer types when they are related. We tried predicting rectal cancer prognosis [28] having trained ENCAPP using colon cancer data [28]. ENCAPP is very successful (median AUC = 0.803; 95% CI for AUC = 0.782-0.823; Figure 5D; *P* < 10^−3^; Additional file 1: Table S1) at predicting rectal cancer prognosis showing that ENCAPP is able to predict prognosis across related cancers.

### Identifying prognostic markers using ENCAPP

**Figure 6:**
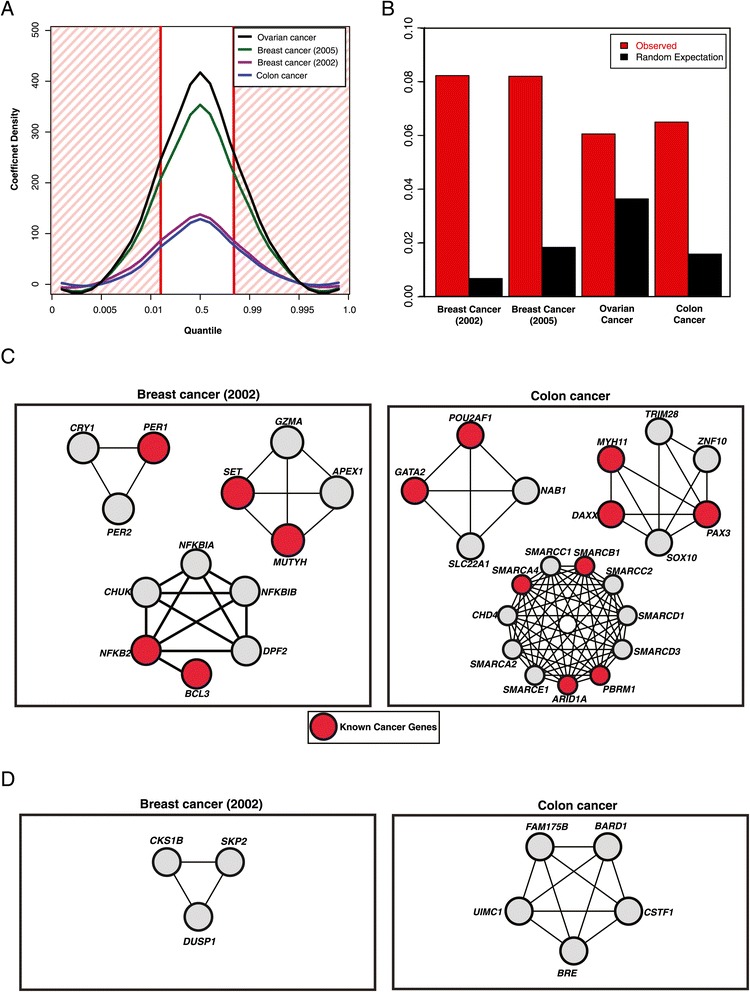
**Prognostic biomarker discovery using ENCAPP. (A)** Distribution of regression coefficients for different human cancers. The red shaded area corresponds to the top 10 percentile. Significant modules are defined as those with coefficients in the red shaded area. **(B)** Enrichment of known cancer genes in the significant modules for the breast cancer (2002), breast cancer (2005), colon cancer and ovarian cancer datasets. **(C)** Examples of significant modules for the breast cancer (2002) and colon cancer datasets. Known cancer genes are depicted in red. **(D)** Examples of novel biomarker prediction for the breast cancer (2002) and colon cancer datasets.

Since our elastic net approach is a combination of LASSO and ridge regression, the number of coefficients with significant regression coefficients is relatively low (Figure 6A, Additional file 3: Table S2; see Methods). The modules whose corresponding coefficients are mathematically significant are termed ‘significant modules’. To test the robustness of these ‘significant’ modules, we calculated the Spearman rank correlation coefficient of these significant modules across cross-validation runs and folds (Additional file 4: Figure S1). We find that they are highly stable: 99.1% have a rank correlation coefficient > = 0.98. To see if these modules are also biologically significant, we examined the distribution of known cancer genes in these modules (see Methods). We found that these modules are significantly enriched for cancer genes (Figure 6B; *P* < 0.01 for all 4 datasets). The fact that the enrichment extends to the level of entire modules shows that the differences in expression patterns extend to the level of the modules themselves. This is conceptually consistent with previous findings that gene sets rather than genes themselves better explain dysregulation in cancer [30]. Thus differential co-expression of these modules is a molecular determinant of different outcomes for different patients.

We also compared the average degree of proteins in these significant modules with that of cancer-associated proteins (11.2) and all proteins in the network (8.2). The average degree of proteins in significant modules is not, in general, skewed towards the average degree of cancer-associated proteins. For the van de Vijver and Wang breast cancer datasets, the average degree of proteins in significant modules are 12.0 and 10.5 respectively, similar to the average degree of cancer-associated proteins. However, for the colon and ovarian cancer datasets, they are 8.3 and 8.8 respectively, similar to the overall average degree. These findings are also consistent with Figure 6B, which shows that the enrichment of cancer genes in significant modules for the 2002 and 2005 breast cancer datasets is higher than the enrichment for the colon and ovarian cancer datasets. This could be due to higher noise for the colon and ovarian cancer datasets or due to the list of cancer genes being incomplete with varying degrees of incompleteness for different tissue types.

To examine whether the significant modules that we find agree with what has been previously reported, we compared the significant modules that we obtained for the van de Vijver dataset with the significant modules that Wu and Stein [14] obtained for the same dataset. 29/85 (34.1%) of the modules are overlapping. Thus, ENCAPP does find a large number of signatures concordant with what has been reported earlier, but it also finds a significant number of potentially novel signatures. We then compared the significant modules that we obtained for the Wang dataset with the significant modules that Wu and Stein obtained for the van de Vijver dataset. This is a comparison both across methods and cancer sub-types. 25/268 (9.3%) of the modules are still overlapping, showing that there are a number of stable signatures across cancer sub-types. We also find that 3 significant modules for the van de Vijver dataset contain 13 proteins of which 5 have been previously implicated in cancer (Figure 6C) and 3 significant modules for the colon cancer dataset contain 9/21 known cancer genes (Figure 6C). A number of these genes are known to be good prognostic markers.

As a further validation, we examined prognostic biomarkers detected by ENCAPP that were unknown at the time of publication of the expression dataset, but have since been clinically validated. Conceptually, these correspond to novel biomarkers detected by ENCAPP. For example, we detected *NFKB2* and *BCL3* in a significant module for the breast cancer (2002) dataset (Figure 6C). In 2005, it was shown that the *NFκB* complex*,* of which *NFKB2* is one of the subunits, can be used a well-known prognostic marker for breast cancer [31]. More recently, it has also been shown that suppression of the *NFκB* co-factor *BCL3* correlates with poor prognosis as it inhibits apoptosis of mammary cells [32]. *GATA2* was present in a significant module for the colon cancer (published in 2011) dataset (Figure 6C). In 2013, *GATA2* was shown to be a useful prognostic marker for colorectal cancer – patients with high expression levels of *GATA2* are likely to have worse disease-free survival outcomes than those with lower expression levels of *GATA2* [33]. These confirm that the significant modules identified by ENCAPP contain numerous prognostic markers.

We also found a number of modules with proteins that have not yet been validated as prognostic biomarkers but are excellent candidates for hypothesis-driven follow-up experiments. For example, one of the significant modules for the breast cancer (2002) dataset contains *CKS1B*, *SKP2* and *DUSP1* (Figure 6D). It has been shown that *CKS1B* is required for the *SKP2*-mediated ubiquitination of *PSMD9* (*p27*) [34]. A recent study shows that *PSMD9* expression is altered in breast cancer patients irrespective of the *BRCA* mutation state [35]. Together, these results suggest that this module and especially *CKS1B* and *SKP2* could be reliable prognostic markers across breast cancer subtypes as altered expression of these genes will lead to mis-regulation of *PSMD9*, whose expression is altered in breast cancer patients with or without mutations in *BRCA1*.

For the colon cancer dataset, one of the significant modules contains *FAM175B*, *BARD1, CSTF1*, *BRE*, and *UIMC1* (Figure 6D). It is well known that *BARD1* interacts with *BRCA1* to form a ubiquitin ligase complex [36,37] and the interaction can be disrupted by breast cancer mutations on *BRCA1* [36,37]. A blood test based on *BARD1* has been proposed as a potential way to diagnose breast cancer [38]. *FAM175B* (*ABRO1*) and *BRE* are two of the 4 subunits of the BRISC deubiquitinating enzyme complex [39]. *BRE* has already been shown to be a reliable prognostic marker for acute myeloid leukemia [40,41]. In the context of these studies, our results suggest that this module and especially *FAM175B*, *BARD1* and *BRE* can be potential prognostic markers for colon cancer as altered expression of these genes can modify ubiquitination activity in the cell.

## Conclusions

Here we have described ENCAPP, a robust prognosis predictor of different human cancers. Since ENCAPP uses differentially expressed modules between patients with good and bad prognosis to accurately predict disease outcome, the decision boundaries used to make this prediction correspond to functional changes in the cell. This is potentially extremely useful in generating mechanistic hypotheses regarding cancer causation and progression that can then be experimentally tested. Conceptually, the ENCAPP algorithm uses interaction dynamics, a combination of gene expression data with the topological structure of the network, to predict prognosis. Previous studies have shown that interaction dynamics is also useful in understanding the organization and evolutionary modes of biological networks [42,43]. Together, these suggest that approaches using interaction dynamics may be successful in elucidating the mechanistic basis of a wide range of biological phenomena, by combining two discrete layers of information – gene expression and protein networks.

Another key feature of ENCAPP is its ability to identify prognostic markers from the regression model itself. While some previous methods show examples of prognostically relevant genes identified by their method [13,15], the key difference is that such detections are typically anecdotal. On the other hand, we demonstrate that the significant modules in ENCAPP are systematically enriched for cancer genes. Thus, our model identifies biologically relevant genes and uses these for determining prognostic outcome. We also show that significant modules identified by ENCAPP contain known prognostic markers and hypothesize that they may contain novel biomarkers. Follow-up studies may want to validate these putative prognostic markers. Since ENCAPP identifies modules containing these genes, any positive results emerging from such studies will directly tie in to a pathway-level understanding of the mechanistic basis of that specific cancer type.

One limitation of ENCAPP is that the accuracy of the prognosis prediction is highly dependent on the quality of the expression dataset, which is why the AUCs vary across the different cancers. Future approaches may want to combine gene expression and protein networks with other data such as somatic mutations, epigenetic modifications and copy number alterations to make the overall prediction accuracy less dependent on the quality of an individual dataset.

## Methods

### Expression data and the human protein interactome network

Sample size, number of good and bad prognosis patients, and breakdown by stage and grade for the different expression datasets used are available in Additional file 5: Table S3. Expression data were RMA-normalized as described in Additional file 2: Supplementary Notes. High-quality binary and co-complex human protein interactome networks were obtained from HINT [16]. The final network used for this study was the union of the binary and co-complex networks. It comprises 42,604 interactions between 9,985 proteins. All datasets used in this study are obtained from papers that have already been published and required no ethics approval.

### Identifying functional modules using clustering

ClusterOne identifies overlapping functional modules based on the topological properties of the protein interactome network [25]. We did a sweep for the ‘*s*’ (size) and ‘*d*’ (minimum cluster density) parameters in ClusterOne [25]. The default parameters are *s* = 3 and *d* = 0.35. We examined the parameter space around these values. Since the modules were identified independently of the expression datasets, situations occasionally arose in which some modules had missing gene expression values. In these cases, a module was included only if at least 1/3 of the genes in that module had corresponding expression values. For each cancer type, we report the highest AUC value obtained in the parameter sweep.

### The elastic-net-based regression model

The elastic net [19] is a regularized regression model that uses a linear combination of the L1 penalty term from LASSO [18] and the L2 penalty term from ridge regression [17]. The objective function is given by:

$$ { \min}_{\beta_0,\beta}\left[{\displaystyle \sum_{\mathrm{i}=1}^N{\left({\mathrm{y}}_{\mathrm{i}}-{\beta}_0-{{\mathrm{x}}_{\mathrm{i}}}^{\mathrm{T}}\beta \right)}^2+\lambda {\mathrm{P}}_{\alpha}\left(\beta \right)}\right] $$

where, *y*_*i*_ corresponds to the prognostic outcome for the *i*^th^ patient (0 or 1 corresponding to good and bad prognosis). *x*_*i*_ is a vector of a vector of features for the *i*^th^ patient (please see below for a detailed description of *x*_*i*_). The β’s are regression coefficients that we estimate. The tuning parameter *λ* is the weight of the regularization term and is chosen to minimize mean square error. The regularization term P_α_(β) is given by:

$$ {\mathrm{P}}_{\alpha}\left(\beta \right)={\displaystyle \sum_{\mathrm{j}=1}^{\left|{\mathrm{x}}_{\mathrm{i}}\right|}\left(\frac{1-\alpha }{2}{\beta_{\mathrm{j}}}^2+\alpha \left|{\beta}_{\mathrm{j}}\right|\right)} $$

Here, α is a number between 0 and 1 with α = 0 corresponding to ridge regression alone and α = 1 corresponding to LASSO alone. We choose the best α using cross validation.

For our first analysis (Figure 2a) that used only expression data, *x*_*i*_ is a vector of dimension *n* containing expression values for *n* genes for the *i*^th^ patient (The entire set of expression values for *d* patients will be a matrix of size *d* x *n*, where each row is the transpose of *x*_*i*_.). For ENCAPP, *x*_*i*_ is a vector of dimension 2*n* containing expression values for *n* modules for the *i*^th^ patient. Each functional module *m* contributes 2 values – *G*_*im*_ and *B*_*im*_ to *x*_*i*_:

$$ {P}_{ik}=\frac{E_{ik}-<{E}_{kg}>}{\sigma_{kg}} $$

$$ {Q}_{ik}=\frac{E_{ik}-<{E}_{kb}>}{\sigma_{kb}} $$

$$ {G}_{im}=\frac{{\displaystyle \sum_{k=1}^{s_m}{P}_{ik}}}{s_m} $$

$$ {B}_{im}=\frac{{\displaystyle \sum_{k=1}^{s_m}{Q}_{ik}}}{s_m} $$

Here, *s*_*m*_ is the number of genes in module *m. E*_*ik*_ corresponds to the expression value of the *k*^th^ gene for the *i*^th^ patient. < *E*_*kg*_ > and < *E*_*kb*_ > represent the average (mean) expression values of the *k*^th^ gene across all patients with good and bad prognosis respectively. *σ*_*kg*_ and *σ*_*kb*_ represent the standard deviation of the expression values of the *k*^th^ gene across all patients with good and bad prognosis respectively. For all the cross-validations, < *E*_*kg*_ >, < *E*_*kb*_ >, *σ*_*kg*_ and *σ*_*kb*_ are calculated using only the samples in the training set. However, while using *G*_*im*_ and *B*_*im*_ as features derived from every module generally gives the most optimum performance, we noticed that in certain cases it is possible to obtain a slight increase in performance by not averaging over each module. There all *P*_*ik*_ and *Q*_*ik*_ values are used as input. While training the ENCAPP classifier, it is necessary to check which of the two approaches performs better.

For the datasets where clinical information was also available, we incorporated it using a logistic regression model. A description of the available clinical data is given in Additional file 6: Clinical information. Since the clinical data is not high dimensional, elastic net regression is not a suitable choice for it. The final predicted outcome was a weighted linear combination of the two outputs – one predicted by the elastic-net-based model (using expression and protein network data) and the other predicted by the logistic regression model. Thus, *Y*_*1*_ = f(*X*_*1*_) and *Y*_*2*_ = g(*X*_*2*_) and *Y* = *k* x *Y*_*1*_ + (1 - *k*) x *Y*_*2*_. Here, *X*_*1*_ is the set of expression derived features, f the function obtained from the elastic-net based classifier and *Y*_*1*_ the corresponding outcome variable, *X*_*2*_ the set of clinical features, f the function obtained from the logistic-regression based classifier and *Y*_*2*_ the corresponding outcome variable. *Y* is the final outcome obtained by a linear combination of *Y*_*1*_ and *Y*_*2*_. An optimal value of *k*, the relative weight parameter is obtained by grid search.

ENCAPP code and associated datasets are available in the Supplementary information (Additional file 7: ENCAPP_Code_Datasets).

### Evaluating performance

The performance of our model is evaluated in a five-fold cross validation framework (Additional file 2: Supplementary Notes). We split the patients into five subsets such that four subsets are used for training and the fifth one is the test set. The prognostic outcomes for the training set were used to determine the regression coefficients. These coefficients were then used to predict outcomes for patients in the test set. We repeated this procedure five times so that each subset served as a test set. The predicted outcomes were compared to the actual outcomes using a receiver operating characteristic (ROC) curve [44]. The area under the ROC curve (AUC) and classification accuracy were used as measure of the quality of the prediction [44]. The cross validation is process was repeated 50 times with a set of random seeds. For all comparisons, each method was run with the same set of random seeds, which ensured that the cross-validation dataset splits were identical across methods. Thus, all observed differences are solely due to one method being superior to the other and not because of how the dataset was split into the 5 folds. *P*-values evaluating the significance of difference in performance between different methods (two sets of AUC values) were calculated using a Mann–Whitney *U* test.

Classification accuracy is measured at the optimum point on the ROC curve. This is usually the point where the slope of the curve (*S*) is given by:

$$ S=\frac{c\left(P\Big|N\right)-c\left(N\Big|N\right)}{c\left(N\Big|P\right)-\mathrm{c}\left(P\Big|P\right)}\times \frac{N}{P} $$

Here, *c*(*I*|*J*) represents the cost of assigning class *I* to class *J*. Here, *P* = true positives + false negatives and *N* = true negatives + false positives are the total counts in the positive and negative classes, respectively. For our calculations, we chose c(*P*|*P*) = c(*N*|*N*) = 0. And c(*N*|*P*) = c(*P*|*N*). Substituting these values, we get,

$$ S=\frac{N}{P} $$

### Enrichment of cancer genes in modules with significant regression coefficients

To identify modules with significant regression coefficients, we examined the distribution of coefficients and chose the highest and lowest two percentile of coefficients as significant (Figure 6A). We then examined the genes in these modules and compared them to known cancer genes. A list of known cancer genes was obtained from the Cancer Gene Census [45]. This is a high-confidence list of manually curated cancer genes with orthogonal layers of evidence, including but not limited to mutation information from COSMIC [46]. The expected fraction of cancer genes identified by random is given by:

$$ {Ef}_i=\frac{C_i}{T_i} $$

where *C*_*i*_ is the number of cancer genes and *T*_*i*_ the total number of genes in modules in the *i*^th^ expression dataset. The observed fraction of cancer genes in modules with significant regression coefficients is given by:

$$ {Of}_i=\frac{X_i}{N_i} $$

where *X*_*i*_ is the actual number of cancer genes and *N*_*i*_ the total number of unique genes in these modules. Thus, the enrichment of cancer genes in modules with significant regression coefficients is given by:

$$ En=\frac{Of_i}{Ef_i} $$

*P*-values were calculated using a cumulative binomial test.

### Availability of supporting data

The datasets supporting the results of this article are included within its additional files.

## Additional files

Additional file 1: Table S1. Summary of AUCs and *p* values for the different datasets. Additional file 2: Supplementary Notes. Supplementary Notes. Additional file 3: Table S2. Number of different modules identified by each clustering method and list of significant modules identified by ENCAPP for the breast cancer (2002), breast cancer (2005), colon cancer and ovarian cancer datasets. All genes in a particular module are listed in a single row. Each module is listed in a separate row. Additional file 4: Figure S1. Distribution of Spearman rank correlation coefficients between significant modules identified across cross validation runs and folds. Additional file 5: Table S3. Sample size, number of good and bad prognosis patients, breakdown by stage and grade for the different datasets. Additional file 6: Clinical information. Description of the clinical information used. Additional file 7: ENCAPP_Code_Datasets. ENCAPP code and associated datasets.
