# Supplementary material for: ENCAPP: elastic-net-based prognosis prediction and biomarker discovery for human cancers
Source: BMC Genomics. 2015 Apr 3;16(1):263. doi: 10.1186/s12864-015-1465-9 (PMC4392808; doi:10.1186/s12864-015-1465-9)
Supplement: Additional file 2: Supplementary Notes. — Supplementary Notes. [file 12864_2015_1465_MOESM2_ESM.pdf]

Supplementary information for:

ENCAPP: elastic-net-based prognosis prediction and biomarker discovery for human cancers

Jishnu Das[1,2*], Kaitlyn M. Gayvert[3*], Florentina Bunea[4], Marten H. Wegkamp[4], Haiyuan Yu[1,2¶]

[1]Department of Biological Statistics and Computational Biology; Cornell University, Ithaca, NY 14853, [2] Weill Institute for Cell and Molecular Biology; Cornell University, Ithaca, NY 14853, [3]Tri-Institutional Training Program in Computational Biology and Medicine, New York, NY 10065, [4]Department of Statistical Science; Cornell University, Ithaca, NY 14853, USA.

*These authors contributed equally to this work. [¶]To whom correspondence should be addressed. Email: haiyuan.yu@cornell.edu

Supplementary Note I: Pre-processing different expression datasets

The different expression datasets were normalized as described below:

<u>Breast cancer – van de Vijver, et al. (2002)</u>

A pre-normalized dataset was used. The expression data was corrected for background level and normalized (please refer to Methods of van de Vijver, et al. 2002)

<u>Breast cancer – Wang, et al. (2005)</u>

Here, the data was obtained in raw .CEL form. We then performed the robust multi-array average (RMA) procedure to normalize the data using Matlab, which has the following steps:

1.Background correction

2. Log2 transformation

3. Quantile normalization

4. Summarization – fitting a linear model to the normalized data in order to obtain an expression measure

<u>Ovarian cancer – The Cancer Genome Atlas (2011)</u>

The ovarian carcinoma dataset was obtained pre-normalized from the TCGA website. This dataset was a compilation of three different platforms: Agilent, Affymetrix HuEx and Affymetrix U133A. The expression data from each array was normalized and summarized separately and then factor analysis was applied to integrate the tree expression values (see Supplementary Methods of Cancer Genome Atlas 2011). After this normalization step was complete, the platinum-resistant patients were extracted to create our final dataset.

<u>Colon cancer – The Cancer Genome Atlas (2012)</u>

The colon cancer dataset came was obtained pre-normalized from the TCGA website. The dataset was originally combined with rectal cancer patients, however those patients were filtered out after the normalization step in order to restrict the dataset to one cancer type. The expression data was measured from a custom Agilent 244K microarray and was normalized as described in the supplementary notes of Cancer Genome Atlas 2012.

Supplementary Note II: Determination of prognostic outcome

For the van de Vijver et al. and TCGA colon and ovarian cancer datasets, good and bad prognostic outcome were defined as patient survival/death within the time frame of the study. For the van de Vijver dataset, the 'death' label was used as the outcome. For the TCGA datasets, the 'vital_status' label was used in place of the 'censored_time_to_death' variable with a time cutoff in order to avoid arbitrarily dichotomizing the prognostic outcomes. Here, this is an appropriate choice given the fact

that the 'censored_time_to_death' variable itself is somewhat arbitrary due to it being measured from the time of diagnosis instead of measured from time of disease onset.

These prognostic outcomes were chosen to be consistent with the outcome used by other methods, in particular to compare the performance on the van de Vijver et al. dataset with the method of Taylor et al. We have also explored how alternate definitions of outcome affect the method performance and found that the results remain relatively consistent. For example, we observed the performance of using patient relapse as the outcome variable for the van de Vijver et al. dataset when we were looking at cross-dataset performance of ENCAPP. In this case, we found that ENCAPP still achieved a strong performance (AUC = 0.72).

For the Wang et al. dataset and the cross-prediction (training on Wang et al, testing on van de Vijver et al) good and bad prognostic outcome were defined as lack of/occurrence of patient relapse within the time frame of the study.

Detailed clinical parameters for all 4 datasets can be found in Table S3.

Supplementary Note III: Cross-validation and performance evaluation

Five-fold cross-validation

For five-fold cross-validation, the entire dataset was randomly divided into five different groups. Four groups were used to train the regression model (and determine the regression coefficients) and the fifth group was used as the test set. This procedure was repeated 5 times so that each group served as the test set once. Since each group served as the test set once, we obtained predictions for all the samples. The predicted values were thresholded at different levels and compared to the true labels to determine the true and false positive rates at each threshold. These true and false positive rates were plotted to generate the ROC curve.

Super-sampling

Both datasets obtained from the TCGA contained a label proportionality problem. In the colon cancer dataset, there were approximately 10 patients with good outcomes to every bad outcome patient. Meanwhile the reverse problem existed in the ovarian carcinoma dataset, with approximately 5 patients with bad outcomes to every good outcome patient. To address this, we introduced super-sampling to even out the number of each outcome in the training set. This was done by duplicating each sample of the under-represented outcome 10/5 times within the training set of each fold of the cross-validation.

Method Comparison

A random seed generator was used to ensure when the performance of different methods were being compared. Each method was run with the same seed, which ensured that the cross-validation dataset splits were identical. Thus, all observed differences are solely due to one method being superior to the other and not because of how the dataset was split into the 5 folds.
