# Supplementary figures and images for: ENCAPP: elastic-net-based prognosis prediction and biomarker discovery for human cancers
Source: BMC Genomics. 2015 Apr 3;16(1):263. doi: 10.1186/s12864-015-1465-9 (PMC4392808; doi:10.1186/s12864-015-1465-9)

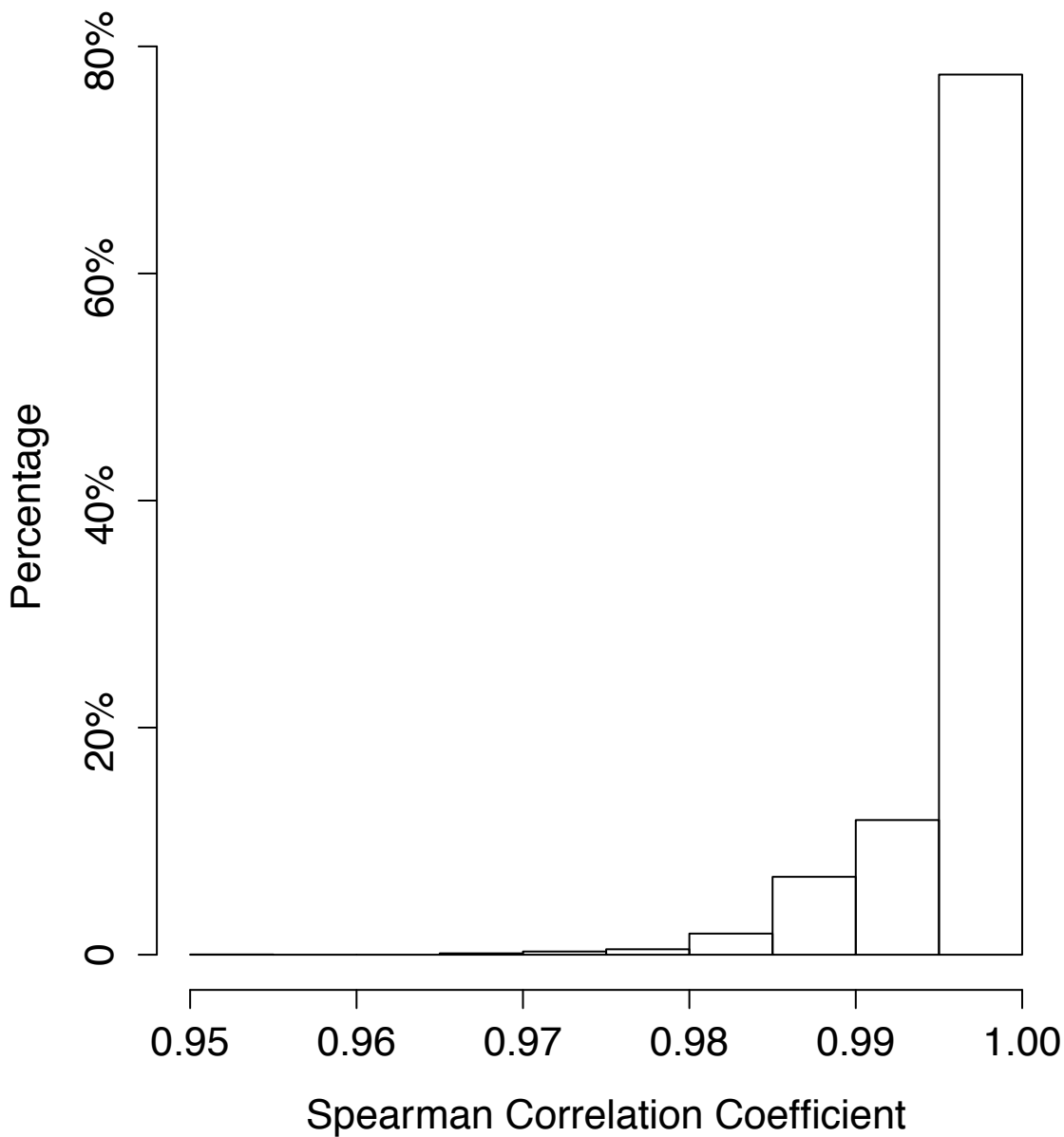

Supplement: Additional file 4: Figure S1. — Distribution of Spearman rank correlation coefficients between significant modules identified across cross validation runs and folds. [file 12864_2015_1465_MOESM4_ESM.pdf]
