# Supplementary material for: ENCAPP: elastic-net-based prognosis prediction and biomarker discovery for human cancers
Source: BMC Genomics. 2015 Apr 3;16(1):263. doi: 10.1186/s12864-015-1465-9 (PMC4392808; doi:10.1186/s12864-015-1465-9)

Summary of Clinical Information Available

van de Vijver et al. (2002) Datasets

The van de Vijver et al. (2002) dataset had a number of clinical features available for each patient. This included tumor diameter (mm), ER status, grade 3 classes, age (years), lymph node (number positive,) and whether chemotherapy and/or hormonal therapy had been given. The breakdown of the above clinical features is broken down below.

Treatment Given	Yes (1)[1]	No (0)
Chemotherapy	110	185
Hormonal Therapy	40	255

ER status	Number of Patients
Positive (1)	226
Negative (0)	69

Grade 3 Classes	Number of Patients
Poorly differentiated (1)	119
Intermediate (2)	101
Well differentiated (3)	75

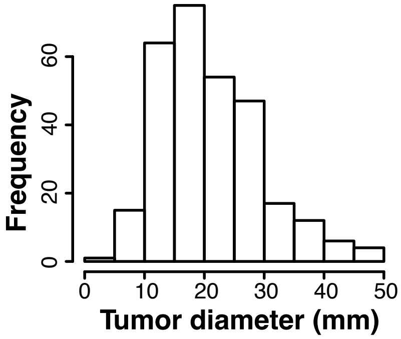

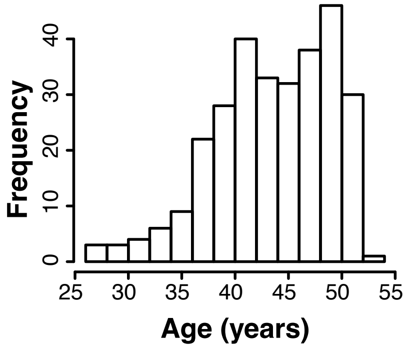

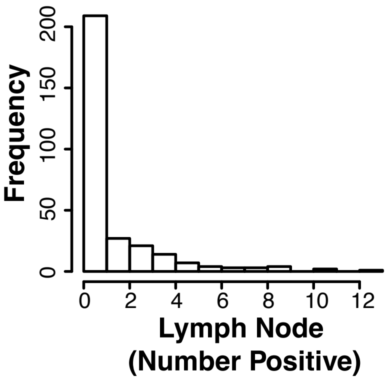

TCGA Colon Cancer (2012) Datasets

The TCGA Colon Cancer (2012) dataset contained the following clinical features for each of its patients: anatomic organ subdivision, tumor site, age at initial pathologic diagnosis, gender, histological type, history of colon polyps, lymphatic invasion present, number of first degree relatives with cancer diagnosis, tumor stage, and vascular invasion present. The breakdown of the spread of these clinical features is broken down below.

Histological Type	Number of Patients
Colon Mucinous Adenocarcinoma (0)[2]	24
Colon Adenocarcinoma (1)	125
Not Available	3

Tumor Site	Number of Patients
right colon (1)	62
transverse colon (2)	14
left colon (3)	71
rectum (4)	1

[1] Note that the number in parentheses for each clinical feature corresponds to the numeric value present in the 2002_clinicalInfo7m.txt file.

[2] Note that the number in parentheses for each clinical feature corresponds to the numeric value present in the ColonDf_clinicalInfo10m.txt file.

**Anatomic Organ Subdivision**	**Number of Patients**
Descending Colon (0)	6
Hepatic Flexure (1)	8
Rectosigmoid Junction (2)	1
Ascending Colon (3)	28
Splenic Flexure (4)	2
Sigmoid Colon (5)	63
Cecum (6)	29
Transverse Colon (7)	14

**Anatomic Organ Subdivision**	**Number of Patients**
Stage I (1)	28
Stage II (2)	60
Stage III (3)	39
Stage IV (4)	23
Not Available	2

	**Yes** (1)	**No** (0)	**Not Available**
**History of Colon Polyps**	78	74	-
**Lymphatic Invasion Present**	77	70	5
**Vascular Invasion Present**	33	105	14

**Number of First Degree Relatives with Cancer Diagnosis**	**Number of Patients**
0	129
1	17
2	4
3	2

**Gender**	**Number of Patients**
Male (1)	77
Female (2)	75

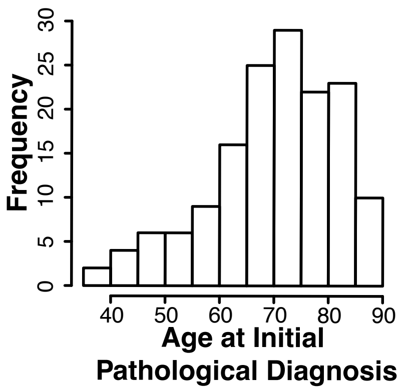

Supplement: Additional file 6: Clinical information. — Description of the clinical information used. [file 12864_2015_1465_MOESM6_ESM.pdf]
